# Divergent and dominant anti-inflammatory effects of patient-derived anticitrullinated protein antibodies (ACPA) in arthritis development

**DOI:** 10.1136/ard-2022-223417

**Published:** 2023-01-05

**Authors:** Bruno Raposo, Marcelo Afonso, Lena Israelsson, Heidi Wähämaa, Ragnhild Stålesen, Fredrik Wermeling, Aase Haj Hensvold, Caroline Grönwall, Bence Rethi, Lars Klareskog, Vivianne Malmström

**Affiliations:** Department of Medicine, Karolinska Universitetssjukhuset i Solna, Stockholm, Sweden

**Keywords:** Anti-Citrullinated Protein Antibodies, Arthritis, Experimental, Arthritis, Rheumatoid, Autoimmunity

**Figure 1:**
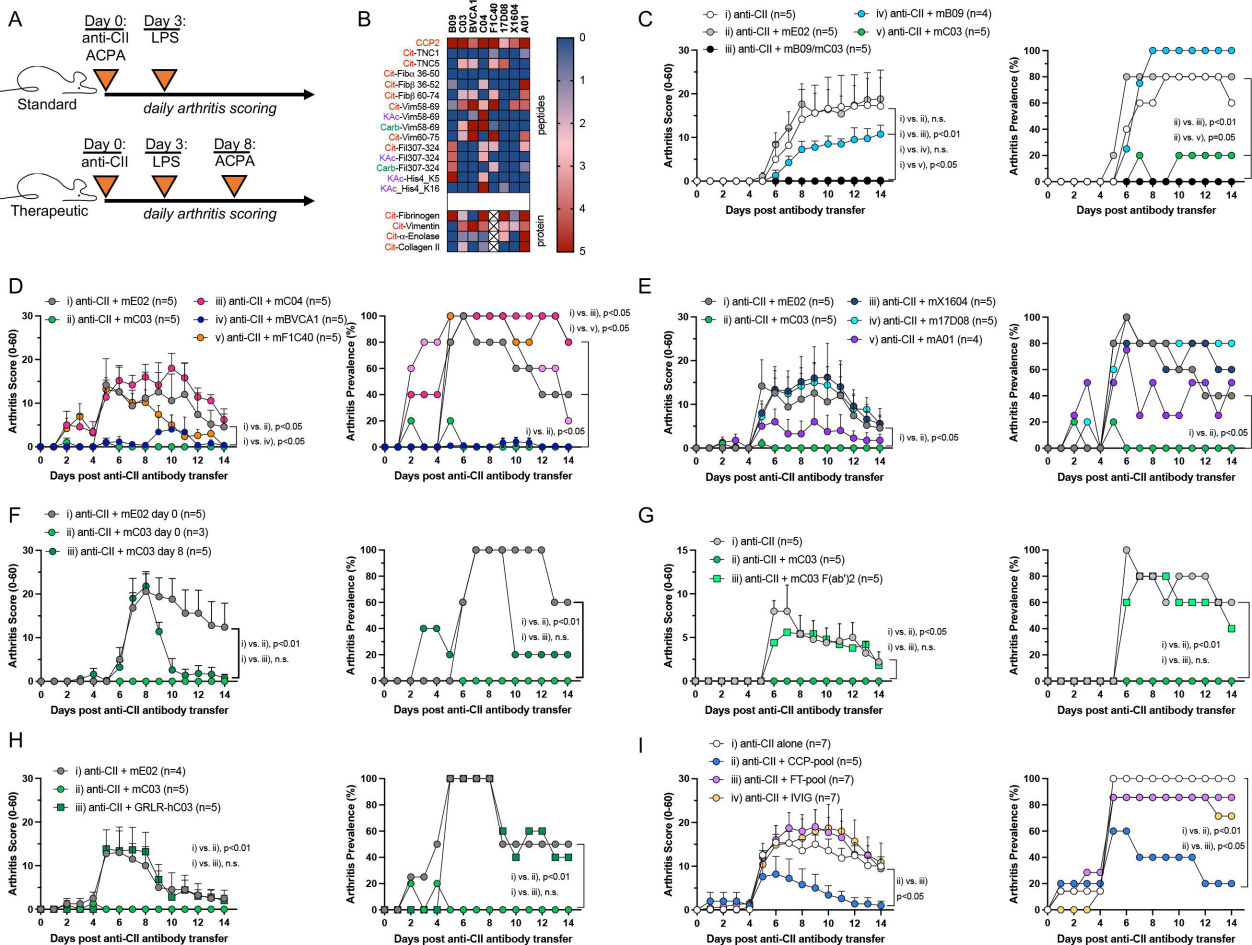
Dominant anti-inflammatory properties evidenced by distinct anti-citrullinated protein antibodies (ACPA) clones in the development of inflammatory arthritis. (A) Schematic representation of the animal model used. (B) Antigen reactivity by monoclonal ACPA used in the study. (C–I) CAIA was induced in mice by intravenous transfer of arthritogenic anti-CII antibody cocktail. Monoclonal ACPA (C, D and E), mC03 F(ab’)2 fragments (G), mutated FcγR-null GRLR-C03 ACPA (H) or polyclonal CCP-pool and respective flow-through (FT) (I) were transferred simultaneously at time of disease induction. Therapeutic effect of ACPA was assessed by transfer of mC03 ACPA at the peak of disease, day 8 (F). Boosting and synchronisation of disease symptoms was done by intraperitoneal administration of LPS 3 days postdisease induction. Statistical analysis calculated by non-parametric repeated-measures Friedman’s test with Dunn’s multiple comparison test. Disease curves from mC03 and mE02 reference groups are identical in C and D due to splitting of the data into two panels for better visualisation. Carb, carbamylation; Cit, citrullination; Fib, fibrinogen; Fil, fillagrin; His4, histone 4; hC03, human IgG1 C0; KAc, acetylated lysine; TNC, tenascin C; Vim, vimentin.

The presence of anti-citrullinated protein antibodies (ACPA) is currently used in rheumatoid arthritis (RA) diagnosis and distinguishes the major subsets of patients. The demonstration that ACPA occur before onset of RA and associate with severe disease^1^ has been used to advocate a causal role of ACPA in disease development. However, not all persistent ACPA-positive individuals progress to clinical RA, suggesting a complex relationship between ACPA and arthritis development, where ACPA displaying different inflammatory aptitudes may exist.^2^ In recent years, we and others have isolated single B cells from RA patients and re-expressed monoclonal ACPA.^3 4^ These monoclonal ACPA display different properties regarding immune-mediated processes in vitro, as well as in vivo phenotypes such as pain and bone erosion.^5 6^ Using the collagen antibody-induced arthritis (CAIA) model of passive arthritis, we assessed the properties of different monoclonal ACPA in vivo concerning their ability to modify the arthritic process (figure 1A). To address this question in an unbiased manner, we tested eight monoclonal ACPA expressed as murine chimeric IgG2a and displaying unique profiles of citrulline-directed fine-specificities (figure 1B and online supplemental figure 1). In line with previous evidence, ACPA per se did not induce arthritis (online supplemental figure 2A). Interestingly, however, we observed several monoclonal ACPA inhibiting (clones mC03 and mBVCA1) or ameliorating arthritis (mB09 and mA01; figure 1C–E), whereas other clones showed no effect in the model (mX1604, m17D08, mF1C40; figure 1 D, E), while one clone provided a slightly enhanced arthritis prevalence (mC04; figure 1D). Using a different model of joint inflammation, the mC04 monoclonal ACPA was previously shown to have an arthritis-accelerating effect.^7^ When ACPA was administered at the peak of disease, mC03-receiving mice recovered almost completely from joint inflammation 48 hours post-ACPA transfer (figure 1F). Similar results were observed with mB09, although less pronounced (online supplemental figure 2B). When combining mC03 and mC04 ACPA, the anti-inflammatory effect of mC03 ACPA prevailed, that is, no arthritis developed (online supplemental figure 2C). The inhibitory effect on arthritis was not linked to any of the known ACPA clones’ fine-specificities, nor to a previously reported histone epitope associated with this effect (online supplemental figure 1E).^2^ The anti-inflammatory effects induced by mC03 were clearly FcγR-dependent, with both its F(ab’)2 fragments and FcγR null (GRLR-mutated) variants incapable of suppressing arthritis development (figure 1G, H; comparison between murine and human C03 ACPA in online supplemental figure 2D). However, no parallel differences in terms of expression of activating or inhibitory FcγR in blood immune cells throughout the course of disease could be observed (online supplemental figure 3). These effects also seemed independent of the ACPA Fc-glycosylation patterns, and the capacity of ACPA in activating the classical complement pathway (online supplemental figure 4). Notably, using IgG ACPA purified by affinity chromatography from a pool of sera from patients with RA, we demonstrate that these polyclonal ACPA seem to be dominantly anti-inflammatory in the CAIA model, contrasting to the respective non-ACPA fraction of the sera (figure 1I).

10.1136/ard-2022-223417.supp1 Supplementary data

The observation that certain ACPA display a beneficial phenotype in inflammatory arthritis needs to be further understood and explored. Whereas some of the here used monoclonal ACPA have previously shown to induce symptoms such as arthralgia, bone loss or tenosynovitis that often precede onset of RA, the present demonstration of a dominant anti-inflammatory effect by certain monoclonal and polyclonal ACPA calls for a re-evaluation of the proposed role of these antibodies in RA. This re-evaluation must consider the heterogeneity of effects mediated by monoclonal ACPA, which together with additional immune stimuli may influence whether an ACPA-positive individual progresses to clinical RA. We acknowledge that the complete molecular mechanism mediating the ACPA anti-inflammatory effects is currently unknown to us and admittedly CAIA is not a citrullination-dependent arthritis model, but the striking effects here shown warrant further investigations. The availability of the monoclonal ACPA used here will enable such urgent investigations to take place.

10.1136/ard-2022-223417.supp2 Supplementary data
